# Association between Triglyceride-Glucose Index and Early Neurological Outcomes after Thrombolysis in Patients with Acute Ischemic Stroke

**DOI:** 10.3390/jcm12103471

**Published:** 2023-05-15

**Authors:** Baixiang Zhang, Hanhan Lei, Gareth Ambler, David J. Werring, Shuangfang Fang, Hangfeng Li, Ronghua Chen, Jin Wei, Guangliang Chen, Nan Liu, Houwei Du

**Affiliations:** 1 Department of Rehabilitation Medicine, Longyan First Affiliated Hospital of Fujian Medical University, Longyan 364099, China; 2 Stroke Research Center, Department of Neurology, Fujian Medical University Union Hospital, Fuzhou 350001, China; 3 Institute of Clinical Neurology, Fujian Medical University, Fuzhou 350005, China; 4 Department of Statistical Science, University College London, London WC1E 6BT, UK; 5 Department of Brain Repair and Rehabilitation, University College London Queen Square Institute of Neurology, London WC1N 3BG, UK; 6 Department of Neurology, Longyan First Affiliated Hospital of Fujian Medical University, Longyan 364099, China; 7 Department of Radiology, Fujian Medical University Union Hospital, Fuzhou 350001, China; 8 Department of Rehabilitation, Fujian Medical University Union Hospital, Fuzhou 350001, China

**Keywords:** acute ischemic stroke, early neurological deterioration, early neurological improvement, intravenous thrombolysis, triglyceride-glucose index

## Abstract

Background: The triglyceride-glucose (TyG) index is a novel biomarker of insulin resistance which might plausibly influence endogenous fibrinolysis and thus early neurological outcomes in patients with acute ischemic stroke (AIS) treated with intravenous thrombolysis using recombinant tissue-plasminogen activator. Methods: We included consecutive AIS patients within 4.5 h of symptom onset undergoing intravenous thrombolysis between January 2015 and June 2022 in this multi-center retrospective observational study. Our primary outcome was early neurological deterioration (END), defined as ≥2 (END_2_) or ≥ 4 (END_4_) National Institutes of Health Stroke Scale (NIHSS) score worsening compared to the initial NIHSS score within 24 h of intravenous thrombolysis. Our secondary outcome was early neurological improvement (ENI), defined as a lower NIHSS score at discharge. TyG index was calculated using the log scale of fasting triglyceride (mg/dL) × fasting glucose (mg/dL)/2. We evaluated the association of END and ENI with TyG index using a logistic regression model. Results: A total of 676 patients with AIS were evaluated. The median age was 68 (Interquartile range, IQR (60–76) years old), and 432 (63.9%) were males. A total of 89 (13.2%) patients developed END_2_, 61 (9.0%) patients developed END_4_, and 492 (72.7%) experienced ENI. In multivariable logistic regression analysis, after adjustment for confounding factors, TyG index was significantly associated with increased risks of END_2_ (categorical variable, vs. lowest tertile, medium tertile odds ratio [OR] 1.05, 95% confidence interval, CI 0.54–2.02, highest tertile OR 2.94, 95%CI 1.64–5.27, overall *p* < 0.001) and END_4_ (categorical variable, vs. lowest tertile, medium tertile OR 1.21, 95%CI 0.54–2.74, highest tertile OR 3.80, 95%CI 1.85–7.79, overall *p* < 0.001), and a lower probability of ENI (categorical variable, vs. lowest tertile, medium tertile OR 1.00, 95%CI 0.63–1.58, highest tertile OR 0.59, 95%CI 0.38–0.93, overall *p* = 0.022). Conclusions: Increasing TyG index was associated with a higher risk of END and a lower probability of ENI in patients with acute ischemic stroke treated with intravenous thrombolysis.

## 1. Introduction

Intravenous thrombolysis treatment remains a first-line approach for acute ischemic stroke (AIS) [1,2]. Despite the beneficial effects of intravenous thrombolysis using recombinant tissue-plasminogen activator for AIS patients, about one-third may experience unfavorable early neurological outcomes [3,4]; 13.8% (95% confidence interval, [CI] 10.0% to 17.7%) of patients experienced early neurological deterioration (END) [5], and 20.9% had failure of early neurological improvement (ENI) [6]. Previous studies showed that END was related to unfavorable stroke outcomes, while ENI was associated with favorable prognosis [6,7,8]. Therefore, identifying factors for early neurological outcomes including END and ENI in the AIS population could provide important prognostic information with relevance for stroke management.

Insulin resistance is considered a main pathophysiological mediator of metabolic syndrome [9]. Despite the established importance of insulin resistance in cardiovascular and cerebrovascular disease [10], evidence of the link between insulin resistance and acute ischemic stroke outcomes is scarce [11]. Insulin resistance might be relevant to acute recanalization therapy through its associations with thrombosis and inflammation, with abnormal endogenous fibrinolysis and increased platelet activation. The hyperinsulinemic-euglycemic clamp test is the gold standard for evaluating insulin resistance. However, this labor-intensive and time-consuming procedure is not routinely measured in clinical practice [12]. Recently, the triglyceride-glucose (TyG) index, which is calculated using serum triglyceride and fast blood glucose levels, has been used as a reliable and novel biomarker of insulin resistance [13]. Aggravating data showed that the TyG index is related to arterial stiffness [14], a higher risk of the cardiocerebrovascular diseases and unfavorable outcomes in patients with cardiocerebrovascular disease [15,16]. Previous studies showed that elevated triglyceride and blood glucose levels were related to the incidence of END and adversely associated with ENI in ischemic stroke population [5,17,18]. Moreover, data from the UK Biobank cohort involving 273,368 individuals showed that the TyG index was superior to blood glucose and triglycerides alone in predicting stroke occurrence, suggesting that the TyG index may potentially be a good biomarker of insulin resistance to predict stroke outcomes [19]. We hypothesized that a higher baseline TyG index is associated with an increased risk of END and a lower probability of ENI in AIS patients who received intravenous thrombolysis. Therefore, we investigated the association of TyG index with the risk of END and ENI after intravenous thrombolysis in a retrospective observational study.

## 2. Methods

### 2.1. Study Design and Participants

We included consecutive adult AIS patients undergoing intravenous thrombolysis within 4.5 h at three certified stroke centers of Fujian Medical University between January 2015 and June 2022 in this multi-center retrospective cohort study. The exclusion criteria were as follows: (1) discharged within 24 h; (2) receiving arterial thrombolytic treatment; (3) interrupted intravenous thrombolysis due to prompt neurological function improvement or serious side effects.

### 2.2. Data Acquisition

Two authors extracted data regarding baseline demographic characteristics, vascular risk factors (smoking, drinking, history of stroke, hypertension, diabetes mellitus, dyslipidemia, atrial fibrillation, and coronary artery disease), pre-stroke medication-use history (antiplatelet, anticoagulation, statin, antihypertensive, and hypoglycemic agents), clinical features (admission stroke onset severity, admission systolic blood pressure and diastolic blood pressure, onset to treatment time, stroke subtypes), and laboratory findings. Stroke severity (presenting deficit severity) was assessed using the National Institutes of Health Stroke Scale (NIHSS) score. Stroke subtypes were classified into atherosclerosis (A), small vessel disease (S), cardioembolic (C), and others (O) [20]. Patients with missing data regarding the component of the TyG index and outcomes were excluded.

### 2.3. Triglyceride Glucose (TyG) Index Evaluation

Blood samples were collected after fasting for 8 to 12 h. Serum triglyceride and glucose levels were assessed with an automatic biochemical analyzer (Cobas c-system, Roche, Switzerland), and expressed in milligrams per deciliter (mg/dL). The TyG index was calculated using the log scale of fasting triglyceride (mg/dL) × fasting glucose (mg/dL)/2, as previously described [21].

### 2.4. Outcomes

Trained clinicians assessed the neurological deficit using the NIHSS score before and after intravenous thrombolysis. Our primary outcome was END. We applied two well-validated (available) definitions: (i) ≥2 NIHSS-point worsening (END_2_); and (ii) ≥4 NIHSS-point worsening (END_4_) compared to the initial NIHSS score within 24 h after intravenous thrombolysis [22,23]. Our secondary outcome was ENI, defined as a lower NIHSS score at discharge [6].

### 2.5. Statistical Analysis

Categorical variables were expressed as absolute counts with percentages. Continuous variables were expressed as means (standard deviation, SD) if normally distributed, or medians (interquartile range, IQR) if not normally distributed. TyG index was treated as both a three-level group (lowest as reference) and a continuous variable. A general linear model and chi-squared test were used to calculate the *p* for trend between variables for continuous and categorical TyG index data, respectively. To summarize the differences in baseline characteristics in patients with and without END or ENI, continuous variables were compared using the Student’s t-test, the Mann–Whitney U-test, analysis of variance or the Kruskal–Wallis test, as appropriate, and categorical variables were compared using the chi-squared test or Fisher’s exact test. We calculated the absolute risks and absolute risk differences between different TyG index tertile groups for early neurological outcomes. Five conventional multivariate adjusted logistic regression models were applied to assess the association of END or ENI with TyG index. Model 1: by incorporating those with *p* < 0.1 for END or ENI in the univariable analysis in addition to age and sex. Model 2: by incorporating the identified suitable minimally sufficient adjustment sets using a directed acyclic graph (DAG) [24] to minimize potential bias from intermediate variables when assessing the effect of the TyG index on END or ENI. Model 3: by incorporating those with *p* < 0.1 in different TyG index tertile groups in addition to age and sex. Model 4: by incorporating baseline NIHSS score and prespecified vascular risk factors (hypertension, atrial fibrillation, and coronary artery disease) that were shown to be related to the odds of END or ENI in addition to age and sex [5,25,26]. Model 5: by incorporating those variables that are associated with both exposure and outcome variables with *p* < 0.1 in the univariable analysis into the multivariable analysis. Considering the components of the TyG index, the fasting blood glucose, history of diabetes dyslipidemia, and previous hypoglycemic use were not simultaneously introduced along with the TyG index into the multivariable analysis. To test whether the effect of TyG index on END or ENI occurrence varied between age group (<65 vs. ≥65 years), sex (male vs. female), diabetes (yes vs. no), and stroke onset severity (baseline NIHSS <6 vs. ≥6), the interaction terms TyG-by-age, TyG-by-sex, TyG-by-NIHSS, and TyG-by-diabetes history were used as covariates. A priori *p*-value < 0.05 was considered significant for interactions. We conducted subgroup analyses, including age (<65 vs. ≥65 years), sex (male vs. female), baseline NIHSS (<6 vs. ≥6), and diabetes (yes vs. no). We conducted a sensitivity analysis limited to patients not receiving thrombectomy treatment after intravenous thrombolysis. Previous studies showed that symptomatic intracerebral hemorrhage (sICH) was a predictor for END, and END after thrombolysis treatment may be caused by sICH occurrence [6,27]. We further conducted a sensitivity analysis by defining END separately from sICH. We also conducted a separate analysis, investigating the relationship between continuous TyG index and continuous delta NIHSS (24 h NIHSS score-baseline NIHSS score). All variables with a *p*-value < 0.05 were considered statistically significant in this study. All statistical analyses were conducted using SPSS version 26.0 (IBM Corp., Armonk, NY, USA).

## 3. Results

### 3.1. Baseline Characteristics

**Figure 1:**
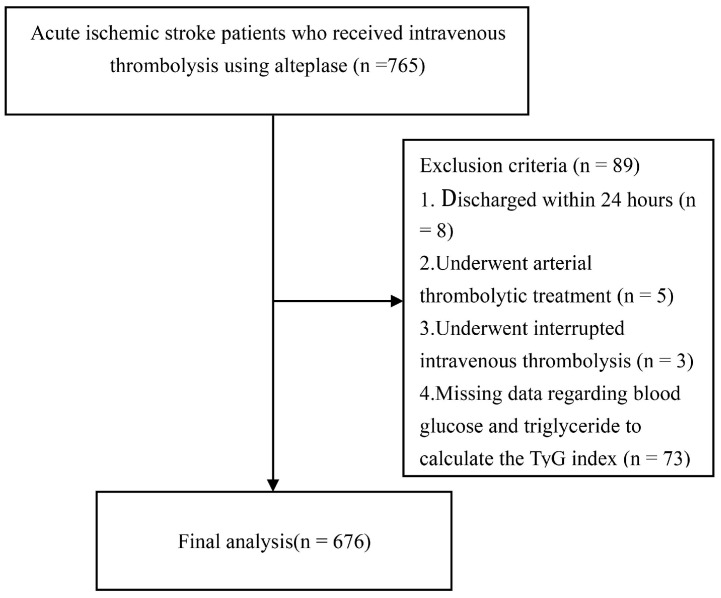
Flow chart. Abbreviations: TyG = Triglyceride-glucose.

From January 2015 through June 2022, a total of 765 adult AIS patients underwent intravenous thrombolysis at three stroke centers (Figure 1). We excluded 89 patients based on the following criteria: eight were discharged within 24 h, five underwent intra-arterial thrombolytic therapy, and three experienced interrupted intravenous thrombolysis; in addition, data of fast glucose and triglyceride to calculate the TyG index were missing in 73 patients. Thus, 676 patients who met the inclusion criteria were included in the final analysis. The median age was 68 [IQR 60–76] years old, and 432 (63.9%) were males. There were no significant differences in median age (68 [IQR 60–76] vs. 65 [IQR 56–76] years) and sex (male: 63.9% vs. 64.8%) between those included and excluded. In those included, stroke onset severity was generally mild to moderate, with a median initial NIHSS score of 6 (IQR 3–12). The median time from symptom onset to thrombolysis treatment was 180 (IQR 120–210) min. The mean value of the TyG index was 8.62 ([SD] 0.70). A total of 28 (4.1%) sICH based on the European Cooperative Acute Stroke Study (ECASS III) definition occurred. A total of 89 (13.2%) patients developed END_2_, 61 (9.0%) developed END_4,_ and 492 (72.7%) experienced ENI at discharge (Table 1). The baseline characteristics of different TyG index groups are summarized in Table 1. Patients with a higher TyG index were more likely to be younger (vs. lowest tertile 70 (62–77), medium tertile 68 (59–75), highest tertile 67 (59–74) years old, overall *p* = 0.064;) and regular alcohol users (vs. lowest tertile 12 (5.3%), medium tertile 24 (10.6%), highest tertile 26 (11.7%), overall *p* = 0.034), and had higher proportions of diabetes mellitus [(vs. lowest tertile 31 (13.7%), medium tertile 48 (21.1%), highest tertile 63 (28.3%), overall *p* < 0.001)] and dyslipidemia [(vs. lowest tertile 42 (18.6%), medium tertile 55 (24.2%), highest tertile 110 (49.3%), overall *p* < 0.001)]. Laboratory findings including fasting blood glucose, triglyceride, total cholesterol, and low-density lipoprotein were significantly higher in patients with a higher TyG index (overall *p* < 0.05).

### 3.2. TyG Index and END

Table 2 summarizes the differences in baseline characteristics in patients with and without END. Patients with END_2_ were older (70 [IQR 62–76] vs. 68 [IQR 59–75], *p* = 0.048), more likely to have atrial fibrillation (49 [55.1%] vs. 161 [27.4%], *p* < 0.001), coronary artery disease (17 [19.1%] vs. 64 [10.9%], *p* = 0.026), and diabetes (27 [30.3%] vs. 115 [19.6%], *p* = 0.020). Pre-stroke hypoglycemic agents (18 [20.2%] vs. 63 [10.7%], *p* = 0.010) were more frequently used in patients with END_2_ compared to those without. Patients with END_2_ more frequently underwent bridging thrombectomy treatment than those without (28 [31.5%] vs. 65 [11.1%], *p* < 0.001). Patients with END_2_ had a higher initial NIHSS score (8 [IQR 4–12] vs. 6 [IQR 3–12], *p* = 0.102), and a higher fast blood glucose level [mg/dl] (121.9 [IQR 95.2–177.7] vs. 101.3 [IQR 87.9–119.2], *p* < 0.001). Stroke subtypes in patients with and without END are significantly different (*p* < 0.001). Similar findings were detected in patients with and without END_4_ (Table 2).

**Figure 2:**
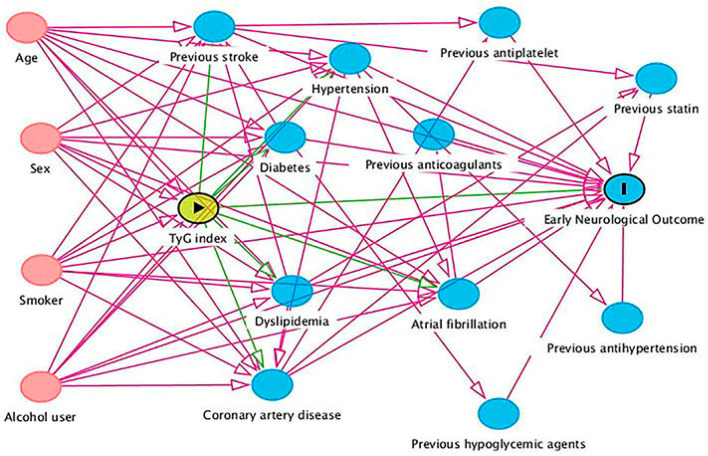
Directed acyclic graph for selection of minimal set of confounders. Abbreviations: TyG = Triglyceride-glucose.

Patients who developed END_2_ included 21 (23.6%) in the lowest tertile (range 6.78–8.30), 21 (23.6%) in the medium tertile (range 8.31–8.86), and 47 (52.8%) in the highest tertile (range 8.89–11.64), compared to 205 (34.9%), 206 (35.1%) and 176 (30.0%), respectively, in those without END_2_ (overall *p* < 0.001). The risk of END_2_ increases with increasing TyG index (continuous variable [per unit increase]; odds ratios: OR 1.77, 95%, CI 1.30–2.42, *p* < 0.001). The absolute risks for END_2_ in the lowest, medium, and highest tertile groups were 9.3%, 9.3%, and 21.1%, respectively. The absolute risk difference for END_2_ (highest vs. lowest) was 11.8% (95% CI, 5.2–18.3%). Similarly, the absolute risks for END_4_ increased with increasing TyG index, while decreasing with increasing TyG index for ENI (Table S1). In the multivariable model 1, adjustment for age, sex, atrial fibrillation, coronary artery disease, initial NIHSS score, ASCO subtypes, and previous stroke use did not change the association between TyG index and the END_2_ risk (categorical variable, vs. lowest tertile, medium tertile OR 1.05, 95% CI 0.54–2.02, highest tertile OR 2.94, 95%CI 1.64–5.27, overall *p* < 0.001; continuous variable [per unit increase], OR 1.91, 95% CI 1.38–2.66, *p* < 0.001). The DAG algorithm identified a minimal set of confounders: age, sex, current smoker, and regular alcohol user (Figure 2). Adjustment for this set did not alter the association between a higher TyG index and an increased risk of END_2_ (Table 3). This association remained in models 3, 4, and 5 (Table S2). We performed a post hoc analysis by combining the lowest and medium groups due to the similar probabilities of END_2_ in these two groups (both 23.6%). The results show that a TyG index in the highest tertile was associated with a higher risk of END_2_ when compared to the lowest and medium groups (adjusted OR 2.87, 95%CI 1.78–4.62, *p* < 0.001, Table 3). Similar findings regarding the association between TyG index and END_4_ risk are shown in Table 4 and Table S3.

A sensitivity analysis limited to patients without endovascular thrombectomy treatment showed that TyG index was associated with both END_2_ (continuous variable [per unit increase], multivariable adjusted OR 1.74, 95%CI 1.19–2.55, *p* = 0.004) (Table S4) and END_4_ risk (continuous variable [per unit increase], multivariable-adjusted OR 1.78, 95%CI 1.10–2.88, *p* = 0.019) (Table S5). However, it was no longer significant when treating TyG index as a categorical variable for the risk of END_4_ (Table S5). Another sensitivity analysis by defining END separately from sICH yielded similar results to those derived from the main analysis (Tables S6 and S7). Subgroup analyses showed that there were no significant interactions between TyG index and variables including age (<65 vs. ≥65 years), diabetes (yes vs. no), and stroke onset severity (baseline NIHSS <6 vs. ≥6) for the risk of END_2_ and END_4_ (all *p* for interaction > 0.05). There was a significant interaction between TyG index and sex (male vs. female) for the risk of END_2_ and END_4_ (Figure S1A,B).

### 3.3. TyG Index and ENI

Differences in baseline characteristics in patients with and without ENI were shown in Table S8. Patients who had ENI included 171 (34.8%) in the lowest tertile, 171 (34.8%) in the medium tertile, and 150 (30.5%) in the highest tertile, compared to 55 (29.9%), 56 (30.4%), and 73 (39.7%), respectively, in those without ENI (overall *p* = 0.077). The odds of ENI decrease with increasing TyG index (continuous variable [per unit increase], OR 0.78, 95% CI 0.61–0.99, *p* = 0.04). In the multivariable model 1, TyG index showed a trend for a lower probability of ENI (categorical variable, vs. lowest tertile, medium tertile OR 1.00, 95%CI 0.63–1.58, highest tertile OR 0.59, 95%CI 0.38–0.92, overall *p* = 0.022; continuous variable [per unit increase], OR 0.73, 95% CI 0.56–0.94, *p* = 0.015). This association remained in other multivariable models (Table S9), but was lost when limited to patients who did not receive endovascular thrombectomy treatment (Table S10). Subgroup analysis showed that there were no significant interactions between the TyG index and variables including age (<65 vs. ≥65 years), sex (male vs. female), diabetes (yes vs. no), and stroke onset severity (baseline NIHSS <6 vs. ≥6) for the odds of ENI (all *p* for interaction > 0.05, Figure S1C).

### 3.4. TyG Index and NIHSS Score Change

Regarding the relationship between continuous TyG index and continuous delta NIHSS (24h NIHSS score-baseline NIHSS score), our data showed that the TyG index was associated with delta NIHSS (unadjusted Beta 0.116, *p* = 0.003, adjusted for the minimal sufficient adjustment set including age, sex, current smoker, and regular alcohol user, adjusted Beta 0.123, *p* = 0.001).

## 4. Discussion

The current study showed that a higher TyG index, a novel biomarker of insulin resistance, is associated with an increased probability of END and decreased odds of ENI in AIS patients who received intravenous thrombolysis; this suggests a role for insulin resistance in the unfavorable early neurological outcome in this population.

The potential role of the TyG index in AIS prognosis has been noted. A multi-center observational study showed that a higher TyG index was associated with 90-day unfavorable functional outcomes in AIS patients who received intravenous thrombolysis [28]. Moreover, TyG index was associated with a higher risk of in-hospital mortality in critically-ill stroke [29], and early stroke recurrence [30]. To our knowledge, whether TyG index is related to unfavorable early neurological functional outcomes is poorly understood. Our data showed that a higher TyG index (particularly being at the top tertile) was associated with a higher probability of developing END and lower odds of achieving ENI at discharge in AIS patients who received intravenous thrombolysis. In line with our finding, a previous study showed that TyG index was associated with END occurrence in patients with single subcortical infarctions [30]. However, that study included AIS patients within 72h of symptom onset applying only one END definition; therefore, the actual frequency of END might be underestimated [30]. Our study adds to previous studies by showing that the association between TyG index and END was consistent when applying two well-validated definitions for END. Moreover, when defining END separately from sICH, the association of END with TyG index remained in our cohort (Tables S6 and S7), since patients who had sICH were older, more likely to have vascular risk factors, higher blood glucose, and a more severe onset (Table S11). Another retrospective cohort study showed that TyG index was inversely associated with ENI (adjusted OR 0.68, 95% CI 0.52–0.89, *p* = 0.004) in AIS patients who received intravenous alteplase thrombolysis [31], which might also support our findings. The aforementioned findings may indicate that the TyG index is probably a biomarker for unfavorable early neurological functional outcomes. It is worth noting that the abovementioned studies were heterogeneous in study population and design; further large-sample size prospective studies are needed to validate the relationship between the TyG index and END risk.

There are several possible explanations for the relationship between TyG index and unfavorable early neurological outcomes after intravenous thrombolysis. First, patients with higher insulin resistance have elevated blood levels of fibrinolysis inhibitors, such as plasminogen activator inhibitors, which may reflect an impairment of endogenous fibrinolytic capacity [32]. Second, insulin resistance is also known to correlate with the worsening response to intravenous thrombolysis [33,34]. Insulin resistance may affect the structure of the offending clot itself, rendering it denser and more resistant to lysis in patients with AIS who received reperfusion treatments [35].

Our findings suggested a sex difference in the association between TyG index and END risk. Possible explanations might include sex disparities in glucose metabolism. For example, impaired glucose tolerance might be more prevalent in women [36]. Moreover, experimental data showed that endogenous estrogen may play a role in higher insulin sensitivity in a female rodent model [37]. Clinical evidence also demonstrated that menopausal hormone therapy may improve insulin sensitivity for postmenopausal women [38]. The above mentioned studies may highlight the need for a sex-specific risk management strategy.

The strengths of our study include using different models to validate the association between TyG index and END risk in a multicenter sample with two-well validated definitions of END. This study has some limitations. First, this is a retrospective observational study with a moderate sample size, inevitably introducing selection bias. Second, this study only included Chinese stroke patients; our findings may therefore not be generalizable to other populations. Third, because of practical limitations derived from our clinical setting, no direct measure to assess insulin resistance was used in our cohort. However, previous studies have shown that the TyG index has high sensitivity and specificity for assessing insulin resistance, suggesting that TyG index could be useful as a surrogate to identify insulin resistance [39]. Lastly, the early measurement may overestimate the prevalence of insulin resistance, since insulin resistance measurement is time-dependent during the acute ischemic stroke onset.

## 5. Conclusions

In conclusion, insulin resistance represented by increased TyG index is associated with higher odds of END and lower odds of ENI in acute ischemic stroke patients who received intravenous thrombolysis. Targeting the TyG index could be a potential obtainable biomarker for risk stratification in this stroke population during routine clinical practice.

## Figures and Tables

**Table 1:** Baseline characteristics of different TyG index groups.

Variable	Total (n = 676)	Tertile I (Lowest) (n = 226)	Tertile II (Medium) (n = 227)	Tertile III (Highest) (n = 223)	*p* for Trend
**Demographic characteristics**					
Age, y, median (IQR)	68 (60–76)	70 (62–77)	68 (59–75)	67 (59–74)	0.064
Sex, n (%)					0.118
Male, n (%)	432 (63.9)	89 (39.4)	83 (36.6)	72 (32.3)	
Female, n (%)	244 (36.1)	137 (60.6)	144 (63.4)	151 (67.7)	
**Vascular risk factors**					
Current smoker, n (%)	198 (29.3)	61 (27.0)	64 (28.2)	73 (32.7)	0.153
Regular alcohol user, n (%)	62 (9.2)	12 (5.3)	24 (10.6)	26 (11.7)	0.034
Previous stroke, n (%)	102 (15.1)	41 (18.1)	33 (14.5)	28 (12.6)	0.098
Hypertension, n (%)	448 (66.3)	143 (63.3)	156 (68.7)	149 (66.8)	0.426
Diabetes, n (%)	142 (21.0)	31 (13.7)	48 (21.1)	63 (28.3)	<0.001
Dyslipidemia, n (%)	207 (30.6)	42 (18.6)	55 (24.2)	110 (49.3)	<0.001
Atrial fibrillation, n (%)	210 (31.1)	78 (34.5)	68 (30.0)	64 (28.7)	0.183
Coronary artery disease, n (%)	81 (12.0)	20 (8.8)	30 (13.2)	31 (13.9)	0.099
**Medication use history**					
Previous antiplatelet, n (%)	75 (11.1)	29 (12.8)	27 (11.9)	19 (8.5)	0.147
Previous anticoagulants, n (%)	14 (2.1)	1 (0.4)	10 (4.4)	3 (1.3)	0.495
Previous statin, n (%)	50 (7.4)	24 (10.6)	15 (6.6)	11 (4.9)	0.021
Previous antihypertension, n (%)	264 (39.1)	85(37.6)	99 (43.6)	80 (35.9)	0.711
Previous hypoglycemic agents, n (%)	81 (12.0)	20 (8.8)	25 (11.0)	36 (16.1)	0.018
**Clinical assessment**					
Initial NIHSS score, median (IQR)	6 (3–12)	7 (3–12)	6 (4–12)	6 (3–12)	0.527
Discharge NIHSS score, median (IQR)	3 (1–7)	3 (1–6)	3 (1–7)	2 (0–7)	0.221
SBP, mmHg, mean ± SD	149 ± 23	150 ± 25	148 ± 22	149 ± 22	0.726
DBP, mmHg, median (IQR)	89 (80–98)	90 (80–98)	87 (80–98)	90 (80–99)	0.285
OTT, minute, median (IQR)	180 (120–210)	180 (120–210)	180 (120–211)	180 (120–210)	0.928
Thrombectomy treatment, n (%)	93 (13.8)	30 (13.3)	33 (14.5)	30 (13.5)	1.000
24 h sICH, n (%)	28 (4.1)	9 (4.0)	4 (1.8)	15 (6.7)	0.147
Any ICH, n (%)	127(18.9)	45 (19.9)	39 (17.2)	43 (19.3)	0.862
**ASCO Stroke subtype**					0.454
Atherosclerosis, n (%)	250 (37.0)	72(31.9)	92 (40.5)	86 (38.6)	
Cardioembolic, n (%)	211 (31.2)	78 (34.5)	70 (30.8)	63 (28.3)	
Small vessel disease, n (%)	70 (10.4)	25 (11.1)	20 (8.8)	25 (11.2)	
Other causes, n (%)	145 (21.4)	51 (22.6)	45 (19.8)	49 (22.0)	
**Laboratory data**					
FBG, mg/dL, median (IQR)	102.9 (88.6–124.3)	93.2 (84.2–107.6)	101.7 (88.7–115.5)	119.3 (100.1–164.8)	<0.001
TG, mg/dL, median (IQR)	99.2 (69.1–144.4)	60.3 (46.0–73.5)	105.4 (88.6–121.3)	174.4 (133.2–231.2)	<0.001
TC, mg/dL, median (IQR)	173.3 (144.4–200.7)	165.4 (132.7–191.0)	168.4 (142.9–195.1)	186.5 (159.4–214.8)	<0.001
LDL, mg/dL, median (IQR)	114.1 (87.1–139.2)	106.2 (78.1–125.7)	114.1 (89.4–136.7)	130.3 (98.1–150.2)	<0.001
**Early Neurological Outcome**					
END_2_	89 (13.2)	21 (9.3)	21 (9.3)	47 (21.1)	<0.001
END_4_	61 (9.0)	13 (5.8)	14 (6.2)	34 (15.2)	<0.001
ENI	492 (72.7)	171 (75.7)	171 (75.3)	150 (67.3)	0.046

Abbreviations: TyG = Triglyceride-glucose; IQR = Interquartile range; NIHSS = National Institute of Health Stroke Scale; SBP = systolic blood pressure; SD = Standard deviation; DBP = diastolic blood pressure; OTT = onset to treatment time; sICH = symptomatic intracerebral hemorrhage; ICH = intracerebral hemorrhage; FBG = fasting blood glucose; TG = triglyceride; TC = total cholesterol; LDL = low-density lipoprotein; END = early neurological deterioration; ENI = early neurological improvement.

**Table 2:** Differences in baseline characteristics in patients with and without END.

Variable	Without END_2_ (n = 587)	With END_2_ (n = 89)	*p* Value	Without END_4_ (n = 615)	With END_4_ (n = 61)	*p* Value
**Demographic characteristics**						
Age, y, median (IQR)	68 (59–75)	70 (62–76)	0.048	68 (59–76)	70 (67–76)	0.019
Sex, n (%)			0.790			0.784
Male, n (%)	374 (63.7)	58 (65.2)		394 (64.1)	38 (62.3)	
Female, n (%)	213 (36.3)	31 (34.8)		221 (35.9)	23 (37.7)	
**Vascular risk factors**						
Current smoker, n (%)	170 (29.0)	28 (31.5)	0.629	181 (29.4)	17 (27.9)	0.798
Regular alcohol user, n (%)	54 (9.2)	8 (9.0)	0.949	59 (9.6)	3 (4.9)	0.228
Previous stroke, n (%)	94 (16.0)	8 (9.0)	0.084	95 (15.4)	7 (11.5)	0.408
Hypertension, n (%)	383 (65.2)	65 (73.0)	0.148	406 (66.0)	42 (68.9)	0.655
Diabetes, n (%)	115 (19.6)	27 (30.3)	0.020	120 (19.5)	22 (36.1)	0.002
Dyslipidemia, n (%)	179 (30.5)	28 (31.5)	0.854	193 (31.4)	14 (23.0)	0.173
Atrial fibrillation, n (%)	161 (27.4)	49 (55.1)	<0.001	168 (27.3)	42 (68.9)	<0.001
Coronary artery disease, n (%)	64 (10.9)	17 (19.1)	0.026	67 (10.9)	14 (23.0)	0.006
**Medication use history**						
Previous antiplatelet, n (%)	63 (10.7)	12 (13.5)	0.441	66 (10.7)	9 (14.8)	0.340
Previous anticoagulants, n (%)	13 (2.2)	1 (1.1)	0.784	13 (2.1)	1 (1.6)	0.804
Previous statin, n (%)	45 (7.7)	5 (5.6)	0.491	46 (7.5)	4 (6.6)	0.793
Previous antihypertension, n (%)	228 (38.8)	36 (40.4)	0.772	239 (38.9)	25 (41.0)	0.746
Previous hypoglycemic agents, n (%)	63 (10.7)	18 (20.2)	0.010	66 (10.7)	15 (24.6)	<0.001
**Clinical assessment**						
Initial NIHSS score, median (IQR)	6 (3–12)	8 (4–12)	0.102	6 (3–12)	12 (7–15)	<0.001
Discharge NIHSS score, median (IQR)	4 (2–9)	16 (8–24)	<0.001	4 (2–9)	18 (15–29)	<0.001
SBP, mmHg, mean ± SD	149 ± 22	151 ± 26	0.621	149 ± 22	151 ± 28	0.476
DBP, mmHg, median (IQR)	89 (80–98)	90 (80–90)	0.533	89 (80–98)	87 (78–99)	0.922
OTT, minute, median (IQR)	180 (120–210)	169 (120–228)	0.513	180 (120–210)	160 (120–220)	0.206
Thrombectomy treatment, n (%)	65 (11.1)	28 (31.5)	<0.001	68 (11.1)	25 (41.0)	<0.001
24 h sICH, n (%)	0 (0.0)	28 (31.5)	<0.001	0 (0.0)	28 (45.9)	<0.001
Any ICH, n (%)	87 (14.8)	40 (44.9)	<0.001	92 (15.0)	45 (57.4)	<0.001
**ASCO Stroke subtype**			<0.001			<0.001
Atherosclerosis, n (%)	222 (37.9)	28 (32.5)		232 (37.7)	18 (29.5)	
Cardioembolic, n (%)	167 (27.3)	44 (50.6)		172 (28.0)	39 (63.9)	
Small vessel disease, n (%)	60 (8.5)	10 (9.6)		67 (10.9)	3 (4.9)	
Other causes, n (%)	138 (26.3)	7 (7.2)		144 (23.4)	1 (1.6)	
**Laboratory data**						
FBG, mg/dL, median (IQR)	101.3 (87.9–119.2)	121.9 (95.2–177.7)	<0.001	100.8 (87.8–119.2)	136.5 (110.3–201.6)	<0.001
TG, mg/dL, median (IQR)	97.5 (69.1–144.4)	103.6 (77.9–143.4)	0.305	99.2 (69.1–147.0)	98.3 (77.0–129.3)	0.712
TC, mg/dL, median (IQR)	173.2 (144.2–200.0)	175.3 (145.4–207.3)	0.643	173.4 (144.7–200.7)	168.6 (138.1–199.9)	0.649
LDL, mg/dL, median (IQR)	113.3 (86.4–138.7)	120.3 (95.1–141.9)	0.178	113.7 (87.0–139.4)	118.3 (92.8–139.2)	0.757
TyG index, mean ± SD	8.58 ± 0.69	8.87 ± 0.68	<0.001	8.59 ± 0.69	8.90 ± 0.68	<0.001
**TyG tertiles**			<0.001			<0.001
Lowest, n (%)	205 (34.9)	21 (23.6)		213 (34.6)	13 (21.3)	
Medium, n (%)	206 (35.1)	21 (23.6)		213 (34.6)	14 (23.0)	
Highest, n (%)	176 (30.0)	47 (52.8)		189 (30.7)	34 (55.7)	

Abbreviations: END = early neurological deterioration; IQR = interquartile range; NIHSS = National Institute of Health Stroke Scale; SBP = systolic blood pressure; SD = standard deviation; DBP = diastolic blood pressure; OTT = onset to treatment time; sICH = symptomatic intracerebral hemorrhage; ICH = intracerebral hemorrhage; FBG = fasting blood glucose; TG = triglyceride; TC = total cholesterol; LDL = low-density lipoprotein; TyG = Triglyceride-glucose.

**Table 3:** Association between TyG index and END_2_.

	Unadjusted		Model 1		Model 2	
	OR (95% CI)	*p* Value	OR (95% CI)	*p* Value	OR (95% CI)	*p* Value
TyG index	1.77 (1.30–2.42)	<0.001	1.91 (1.38–2.66)	<0.001	1.88 (1.37–2.58)	<0.001
**TyG tertiles**		<0.001		<0.001		<0.001
Lowest	Ref		Ref		Ref	
Medium	1.00 (0.53–1.88)		1.05 (0.54–2.02)		1.06 (0.56–2.02)	
Highest	2.61 (1.50–4.53)		2.94 (1.64–5.27)		2.82 (1.61–4.95)	
**TyG binary classification**						
Lowest to medium	Ref		Ref		Ref	
Highest	2.61 (1.66–4.11)	<0.001	2.87 (1.78–4.62)	<0.001	2.74 (1.73–4.33)	<0.001

Model 1 = adjusted for age, sex, atrial fibrillation, coronary artery disease, initial NIHSS score, ASCO subtypes, and previous stroke; Model 2 = adjusted for the minimally sufficient adjustment sets using a directed acyclic graph: age, sex, current smoker, regular alcohol user. Abbreviations: TyG = The triglyceride-glucose; END = early neurological deterioration; OR = odds ratios; CI = confidence interval; sICH = symptomatic intracerebral hemorrhage; NIHSS = National Institute of Health Stroke Scale. ASCO: A (atherosclerosis) S (small vessel disease) C (cardioembolic) O (other causes).

**Table 4:** Association between TyG index and END_4_.

	Unadjusted		Model 1		Model 2	
	OR (95% CI)	*p* Value	OR (95% CI)	*p* Value	OR (95% CI)	*p* Value
TyG index	1.80 (1.26–2.57)	0.001	2.07 (1.40–3.07)	<0.001	1.99 (1.37–2.87)	<0.001
**TyG tertiles**		<0.001		<0.001		<0.001
Lowest	Ref		Ref		Ref	
Medium	1.08 (0.49–2.35)		1.21 (0.54–2.74)		1.20 (0.55–2.62)	
Highest	2.95 (1.51–5.75)		3.80 (1.85–7.79)		3.37 (1.71–6.65)	
**TyG binary classification**						
Lowest to medium	Ref		Ref		Ref	
Highest	2.84 (1.67–4.84)	<0.001	3.45 (1.94–6.12)	<0.001	3.08 (1.79–5.30)	<0.001

Model 1 = adjusted for age, sex, atrial fibrillation, coronary artery disease, initial NIHSS score, ASCO subtypes; Model 2 = adjusted for the minimally sufficient adjustment sets using a directed acyclic graph: age, sex, current smoker, regular alcohol user. Abbreviations: TyG = Triglyceride-glucose; END = early neurological deterioration; OR = odds ratios; CI = confidence interval; sICH = symptomatic intracerebral hemorrhage; NIHSS = National Institute of Health Stroke Scale; ASCO: A (atherosclerosis) S (small vessel disease) C (cardioembolic) O (other causes).

## Data Availability

Data are available from the corresponding author on reasonable request.

## Supplementary Materials

The following supporting information can be downloaded at: https://www.mdpi.com/article/10.3390/jcm12103471/s1, Figure S1: Interactions between the TyG index and END or ENI; Table S1: Absolute risks for early neurological outcomes in different TyG index tertile groups; Table S2: Association between TyG index and END_2_; Table S3: Association between TyG index and END_4_; Table S4: Association between TyG index and END_2_ by limited to patients who did not receive thrombectomy treatment; Table S5: Association between TyG index and END4 by limited to patients who did not receive thrombectomy treatment; Table S6: Association between TyG index and END2 apart from sICH; Table S7: Association between TyG index and END_4_ apart from sICH; Table S8: Differences in baseline characteristics in patients with and without ENI; Table S8: Differences in baseline characteristics in patients with and without ENI; Table S10: Association between TyG index and ENI by limited to patients who did not receive thrombectomy treatment; Table S11: Baseline characteristics in patients with and without early sICH.

## Abbreviations

TyG: Triglyceride-glucose; AIS: acute ischemic stroke; END: early neurological deterioration; ENI: early neurological improvement; NIHSS: National Institutes of Health Stroke Scale; SD: Standard deviation; IQR: Interquartile range; DAG: directed acyclic graph; OR: odds ratios; CI: confidence interval; ASCO: A (atherosclerosis) S (small vessel disease) C (cardioembolic) O (other causes). sICH: symptomatic intracerebral hemorrhage; ECASS: European Cooperative Acute Stroke Study.
