# Supplementary material for: Association between Triglyceride-Glucose Index and Early Neurological Outcomes after Thrombolysis in Patients with Acute Ischemic Stroke
Source: J Clin Med. 2023 May 15;12(10):3471. doi: 10.3390/jcm12103471 (PMC10219034; doi:10.3390/jcm12103471)
Supplement: Supplementary file 1 [file jcm-12-03471-s001.zip › jcm-2174811-supplementary.pdf]

Supplementary Materials

Table S1. Absolute risks for early neurological outcomes in different TyG index tertile groups.

Table S2. Association between TyG index and END_2

Table S3. Association between TyG index and END_4

Table S4. Association between TyG index and END_2 by limited to patients who did not receive thrombectomy treatment

Table S5. Association between TyG index and END_4 by limited to patients who did not receive thrombectomy treatment

Table S6. Association between TyG index and END_2 apart from sICH

Table S7. Association between TyG index and END_4 apart from sICH

Table S8. Differences in baseline characteristics in patients with and without ENI

Table S9. Association between TyG index and ENI

Table S10. Association between TyG index and ENI by limited to patients who did not receive thrombectomy treatment

Table S11. Baseline characteristics in patients with and without early sICH

Figure S1. Interactions between the TyG index and END or ENI.

Figure legend: A: END_2; B: END_4; C: ENI;

Table S1. Absolute risks for early neurological outcomes in different TyG index tertile groups.

TyG tertiles	With END_2	Without END_2	Total	Absolute risk	Absolute risk difference (95% CI %)
Lowest	21	205	226	9.3%	Ref
Medium	21	206	227	9.3%	0.4 (-3.9 to 4.8)
Highest	47	176	223	21.1%	11.8 (5.2 to 18.3);
**TyG tertiles**	**With END_4**	**Without END_4**	**Total**	**Absolute risk**	**Absolute risk difference (95% CI %)**
Lowest	13	189	226	5.8%	Ref
Medium	14	213	227	6.2%	0.4 (-3.9 to 4.8)
Highest	34	213	223	15.2%	9.5 (3.9 to15.1)
**TyG tertiles**	**With ENI**	**Without ENI**	**Total**	**Absolute risk**	**Absolute risk difference 95% CI (%)**
Lowest	171	73	226	75.5%	Ref
Medium	171	56	227	75.3%	0.35 (-7.6 to 8.3)
Highest	150	55	223	67.3%	0.4 (-3.9 to 4.8)

Abbreviations: TyG = Triglyceride-glucose; END = Early neurological deterioration; ENI = Early neurological improvement; CI = confidence interval;

Table S2. Association between TyG index and END2

	Model 3		Model 4		Model 5	
	OR (95% CI)	p value	OR (95% CI)	p value	OR (95% CI)	p value
TyG index	1.82 (1.32-2.51)	<0.001	1.92 (1.39-2.66)	<0.001	1.92 (1.38-2.66)	<0.001
**TyG tertiles**		<0.001		<0.001		<0.001
Lowest	Ref		Ref		Ref	
Medium	1.00 (0.52-1.90)		1.04 (0.54-2.00)		1.03 (0.53-1.99)	
Highest	2.62 (1.49-4.62)		2.95 (1.65-5.26)		2.88 (1.61-5.17)	
**TyG binary classification**						
Lowest to medium	Ref		Ref		Ref	
Highest	2.63 (1.66-4.16)	<0.001	2.89 (1.80-4.64)	<0.001	2.84 (1.77-4.58)	<0.001

Model 3 = adjusted for age, sex, regular alcohol user, previous stroke; coronary artery disease and statin use;

Model 4 = adjusted for age, sex, hypertension, atrial fibrillation, coronary artery disease, initial NIHSS score;

Model 5 = adjusted for age, sex, regular alcohol user, statin use, atrial fibrillation, coronary artery disease, initial NIHSS score, and ASCO subtypes and previous stroke;

Abbreviations: TyG = The triglyceride-glucose; END = Early neurological deterioration; OR = odds ratios; CI = confidence interval; sICH = symptomatic intracerebral hemorrhage; NIHSS = National Institute of Health Stroke Scale; ASCO: A (atherosclerosis) - S (small vessel disease) - C (cardioembolic)- O (other causes).

Table S3. Association between TyG index and END$_4$

	**Model 3**		**Model 4**		**Model 5**	
	OR (95% CI)	p value	OR (95% CI)	p value	OR (95% CI)	p value
TyG index	1.93 (1.33-2.80)	0.001	2.07 (1.40-3.05)	<0.001	2.07 (1.39-3.07)	<0.001
**TyG tertiles**		0.001		<0.001		<0.001
Lowest	Ref		Ref		Ref	
Medium	1.12 (0.51-2.47)		1.26 (0.55-2.85)		1.19 (0.53-2.70)	
Highest	3.11 (1.57-6.16)		3.87 (1.88-7.94)		3.65 (1.78-7.49)	
**TyG binary classification**						
Lowest to medium	Ref		Ref		Ref	
Highest	2.93 (1.70-5.05)	<0.001	3.44 (1.94-6.11)	<0.001	3.34 (1.88-5.94)	<0.001

Model 3=adjusted for age, sex, regular alcohol user, previous stroke; coronary artery disease and statin use;

Model 4=adjusted for age, sex, hypertension, atrial fibrillation, coronary artery disease, initial NIHSS score;

Model 5=adjusted for age, sex, regular alcohol user, previous stroke, statin use, atrial fibrillation, coronary artery disease, initial NIHSS score, and ASCO subtypes;

Abbreviations: TyG = Triglyceride-glucose; END = Early neurological deterioration; OR = odds ratios; CI = confidence interval; sICH = symptomatic intracerebral hemorrhage; NIHSS = National Institute of Health Stroke Scale; ASCO: A (atherosclerosis) - S (small vessel disease) - C (cardioembolic)- O (other causes).

Table S4. Association between TyG index and END_2 by limited to patients who did not receive thrombectomy treatment

	Unadjusted		Model 1		Model 2		Model 3		Model 4		Model 5	
	OR (95% CI)	p value	OR (95% CI)	p value	OR (95% CI)	p value	OR (95% CI)	p value	OR (95% CI)	p value	OR (95% CI)	p value
TyG index	1.63 (1.14-2.34)	0.008	1.74 (1.19-2.55)	0.004	1.72 (1.00-1.06)	0.004	1.68 (1.15-2.44)	0.007	1.74 (1.20-2.54)	0.004	1.73 (1.18-2.53)	0.005
**TyG tertiles**		0.044		0.029		0.033		0.049		0.025		0.035
Lowest	Ref		Ref		Ref		Ref		Ref		Ref	
Medium	1.01 (0.49-2.08)		1.02 (0.48-2.15)		1.08 (0.52-2.23)		0.98 (0.47-2.05)		1.00 (0.48-2.09)		0.98 (0.47-2.08)	
Highest	1.99 (1.04-3.80)		2.14 (1.09-4.20)		2.14 (1.11-4.12)		1.96 (1.01-3.79)		2.14 (1.10-4.17)		2.07 (1.05-4.06)	
**TyG binary classification**												
Lowest to medium	Ref		Ref		Ref		Ref		Ref		Ref	
Highest	1.98 (1.16-3.38)	0.013	1.12 (1.22-3.70)	0.008	2.06 (1.20-3.55)	0.009	1.98 (1.15-3.40)	0.014	2.15 (1.24-3.72)	0.007	2.09 (1.20-3.64)	0.010

Model 1=adjusted for age, sex, atrial fibrillation, coronary artery disease, initial NIHSS score, ASCO subtypes, and previous stroke;

Model 2=adjusted for the minimally sufficient adjustment sets using a directed acyclic graph: age, sex, current smoker, regular alcohol user;

Model 3 = adjusted for age, sex, regular alcohol user, previous stroke; coronary artery disease and statin use;

Model 4 = adjusted for age, sex, hypertension, atrial fibrillation, coronary artery disease, initial NIHSS score;

Model 5 = adjusted for age, sex, regular alcohol user, statin use, atrial fibrillation, coronary artery disease, initial NIHSS score, and ASCO subtypes and previous stroke;

Abbreviations: TyG = Triglyceride-glucose; END = Early neurological deterioration; OR = odds ratios; CI = confidence interval; sICH = symptomatic intracerebral hemorrhage; NIHSS = National Institute of Health Stroke Scale; ASCO: A (atherosclerosis) - S (small vessel disease) - C (cardioembolic)- O (other causes).

Table S5. Association between TyG index and END_4 by limited to patients who did not receive thrombectomy treatment

	Unadjusted		Model 1		Model 2		Model 3		Model 4		Model 5	
	OR (95% CI)	p value	OR (95% CI)	p value	OR (95% CI)	p value	OR (95% CI)	p value	OR (95% CI)	p value	OR (95% CI)	p value
TyG index	1.62 (1.04-2.54)	0.034	1.78 (1.10-2.88)	0.019	1.72 (1.19-2.48)	0.004	1.76 (1.10-2.83)	0.019	1.78 (1.10-2.87)	0.018	1.79 (1.10-2.91)	0.02
**TyG tertiles**		0.182		0.119		0.033		0.166		0.174		0.139
Lowest	Ref		Ref		Ref		Ref		Ref		Ref	
Medium	1.13 (0.45-2.84)		1.21 (0.46-3.18)		1.08 (0.52-2.23)		1.11 (0.44-2.85)		1.21 (0.47-3,11)		1.19 (0.45-3.13)	
Highest	2.01 (0.87-4.62)		2.32 (0.96-5.60)		2.14 (1.11-4.12)		2.06 (0.88-4.83)		2.12 (0.90-5.00)		2.25 (0.93-5.44)	
**TyG binary classification**												
Lowest to medium	Ref		Ref		Ref		Ref		Ref		Ref	
Highest	1.89 (0.96-3.72)	0.067	2.11 (1.03-4.32)	0.042	2.06 (1.20-3.55)	0.009	1.94 (0.97-3.88)	0.059	2.12 (1.04-4.32)	0.04	2.06 (1.00-4.23)	0.050

Model 1=adjusted for age, sex, atrial fibrillation, coronary artery disease, initial NIHSS score, ASCO subtypes;

Model 2=adjusted for the minimally sufficient adjustment sets using a directed acyclic graph: age, sex, current smoker, regular alcohol user;

Model 3=adjusted for age, sex, regular alcohol user, previous stroke; coronary artery disease and statin use;

Model 4=adjusted for age, sex, hypertension, atrial fibrillation, coronary artery disease, initial NIHSS score;

Model 5=adjusted for age, sex, regular alcohol user, previous stroke, statin use, atrial fibrillation, coronary artery disease, initial NIHSS score, and ASCO subtypes;

Abbreviations: TyG = Triglyceride-glucose; END = Early neurological deterioration; OR = odds ratios; CI = confidence interval; sICH = symptomatic intracerebral hemorrhage; NIHSS = National Institute of Health Stroke Scale; ASCO: A (atherosclerosis) - S (small vessel disease) - C (cardioembolic)- O (other causes).

Table S6. Association between TyG index and END_2 apart from sICH

	Unadjusted		Model 1		Model 2		Model 3		Model 4		Model 5	
	OR (95% CI)	p value	OR (95% CI)	p value	OR (95% CI)	p value	OR (95% CI)	p value	OR (95% CI)	p value	OR (95% CI)	p value
TyG index	1.76 (1.23-2.52)	0.002	1.80 (1.23-2.62)	0.002	1.79 (1.24-2.58)	0.002	1.74 (1.20-2.51)	0.003	1.85 (1.27-2.70)	0.001	1.81 (1.23-2.64)	0.002
**TyG tertiles**		0.002		0.003		0.002		0.003		0.002		0.003
Lowest	Ref		Ref		Ref		Ref		Ref		Ref	
Medium	1.41 (0.66-3.03)		1.42 (0.65-3.10)		1.48 (0.69-3.19)		1.39 (0.64-3.02)		1.41 (0.65-3.06)		1.41 (0.64-3.09)	
Highest	3.11 (1.55-6.21)		3.16 (1.54-6.50)		3.26 (1.61-6.59)		3.03 (1.49-6.15)		3.25 (1.59-6.64		3.15 (1.53-6.47)	
**TyG binary classification**												
Lowest to medium	Ref		Ref		Ref		Ref		Ref		Ref	
Highest	2.58 (1.51-4.39)	<0.001	2.60 (1.49-4.53)	0.001	2.63 (1.53-4.52)	<0.001	2.52 (1.47-4.33)	0.001	2.69 (1.56-4.66)	<0.001	2.60 (1.49-4.53)	0.001

Model 1=adjusted for age, sex, atrial fibrillation, coronary artery disease, initial NIHSS score, ASCO subtypes, and previous stroke;

Model 2=adjusted for the minimally sufficient adjustment sets using a directed acyclic graph: age, sex, current smoker, regular alcohol user;

Model 3 = adjusted for age, sex, regular alcohol user, previous stroke; coronary artery disease and statin use;

Model 4 = adjusted for age, sex, hypertension, atrial fibrillation, coronary artery disease, initial NIHSS score;

Model 5 = adjusted for age, sex, regular alcohol user, statin use, atrial fibrillation, coronary artery disease, initial NIHSS score, and ASCO subtypes and previous stroke;

Abbreviations: TyG = Triglyceride-glucose; END = Early neurological deterioration; OR = odds ratios; CI = confidence interval; sICH = symptomatic intracerebral hemorrhage; NIHSS = National Institute of Health Stroke Scale; ASCO: A (atherosclerosis) - S (small vessel disease) - C (cardioembolic)- O (other causes).

Table S7. Association between TyG index and END_4 apart from sICH

	Unadjusted		Model 1		Model 2		Model 3		Model 4		Model 5	
	OR (95% CI)	p value	OR (95% CI)	p value	OR (95% CI)	p value	OR (95% CI)	p value	OR (95% CI)	p value	OR (95% CI)	p value
TyG index	1.85 (1.17-2.93)	0.009	2.05 (1.25 -3.36)	0.005	1.79 (1.24-2.58)	0.002	1.91 (1.19-3.07)	0.007	2.09 (1.28-3.42)	0.003	2.06 (1.25-3.38)	0.005
**TyG tertiles**		0.006		0.002		0.002		0.005		0.002		0.002
Lowest	Ref		Ref		Ref		Ref		Ref		Ref	
Medium	2.50 (0.77-8.10)		2.64 (0.79-8.80)		1.48 (0.69-3.19)		2.60 (0.79-8.52)		2.87 (0.86-9.60)		2.72 (0.81-9.07)	
Highest	5.35 (1.79-16.02)		6.87 (2.19- 21.52)		3.26 (1.61-6.59)		5.61 (1.85-17.04)		6.95 (2.23-21.73)		6.55 (2.11-20.34)	
**TyG binary classification**												
Lowest to medium	Ref		Ref		Ref		Ref		Ref		Ref	
Highest	3.06 (1.50-6.23)	0.002	3.60 (1.71-7.58)	0.001	2.63 (1.53-4.52)	<0.001	3.12 (1.52-6.43)	0.002	3.65 (1.73-7.71)	0.001	3.57 (1.69-7.53)	0.001

Model 1=adjusted for age, sex, atrial fibrillation, coronary artery disease, initial NIHSS score, ASCO subtypes;

Model 2=adjusted for the minimally sufficient adjustment sets using a directed acyclic graph: age, sex, current smoker, regular alcohol user;

Model 3=adjusted for age, sex, regular alcohol user, previous stroke; coronary artery disease and statin use;

Model 4=adjusted for age, sex, hypertension, atrial fibrillation, coronary artery disease, initial NIHSS score;

Model 5=adjusted for age, sex, regular alcohol user, previous stroke, statin use, atrial fibrillation, coronary artery disease, initial NIHSS score, and ASCO subtypes;

Abbreviations: TyG = Triglyceride-glucose; END = Early neurological deterioration; OR = odds ratios; CI = confidence interval; sICH = symptomatic intracerebral hemorrhage; NIHSS = National Institute of Health Stroke Scale; ASCO: A (atherosclerosis) - S (small vessel disease) - C (cardioembolic)- O (other causes).

Table S8. Differences in baseline characteristics in patients with and without ENI

Variable	Without ENI (n = 184)	With ENI (n = 492)	P Value
**Demographic characteristics**			
Age, y, median (IQR)	69 (63-76)	68 (59-75)	0.053
Sex, n (%)			0.519
Male, n (%)	114 (62.0)	318 (64.6)	
Female, n (%)	70 (38.0)	174 (35.4)	
**Vascular risk factors**			
Current smoker, n (%)	40 (21.7)	158 (32.1)	0.008
Regular alcohol user, n (%)	15 (8.2)	47 (9.6)	0.574
Previous stroke, n (%)	25 (13.6)	77 (15.7)	0.505
Hypertension, n (%)	130 (70.7)	318 (64.6)	0.141
Diabetes, n (%)	41 (22.3)	101 (20.5)	0.618
Dyslipidemia, n (%)	65 (35.3)	141 (28.9)	0.105
Atrial fibrillation, n (%)	71 (38.6)	139 (28.3)	0.010
Coronary artery disease, n (%)	26 (14.1)	55 (11.2)	0.293
**Medication use history**			
Previous antiplatelet, n (%)	25 (13.6)	50 (10.2)	0.207
Previous anticoagulants, n (%)	4 (2.2)	10 (2.0)	0.909
Previous statin, n (%)	14 (7.6)	36 (7.3)	0.897
Previous antihypertension, n (%)	75 (40.8)	189 (38.4)	0.578

Previous hypoglycemic agents, n (%)	25 (13.6)	56 (11.4)	0.432
**Clinical assessment**			
Initial NIHSS score, median (IQR)	5 (2-12)	7 (4-12)	<0.001
Discharge NIHSS score, median (IQR)	5 (2-15)	4 (2-10)	0.041
SBP, mmHg, mean±SD	153±23	147±22	0.013
DBP, mmHg, median (IQR)	90 (80-100)	80 (88-98)	0.033
OTT, minute, median (IQR)	180 (136-220)	180 (120.0-210)	0.050
Thrombectomy treatment, n (%)	22 (12.0)	71 (14.4)	0.406
24hours sICH, n (%)	18 (9.8)	10 (2.0)	<0.001
Any ICH, n (%)	40 (21.7)	87 (17.7)	0.229
**ASCO Stroke subtype**			0.400
Atherosclerosis, n (%)	68 (37.0)	182 (37.0)	
Cardioembolic, n (%)	64 (34.8)	147 (29.9)	
Small vessel disease, n (%)	14 (20.7)	107 (21.7)	
Other causes, n (%)	38 (7.6)	56 (11.4)	
**Laboratory data**			
FBG, mg/dl, median (IQR)	108.1 (91.2-141.6)	101.4 (87.2-119.6)	0.001
TG, mg/dl, median (IQR)	103.6 (72.6-142.5)	97.0 (69.1-145.3)	0.512
TC, mg/dl, median (IQR)	175.4 (147.7-207.1)	172.8 (142.0-198.0)	0.133
LDL, mg/dl, median (IQR)	118.6 (92.6-144.8)	111.7 (85.9-138.4)	0.043
TyG index, mean±SD	8.71±0.71	8.58±0.69	0.025
**TyG tertiles**			0.077

Lowest, n (%)	55 (29.9)	171 (34.8)
Medium, n (%)	56 (30.4)	171 (34.8)
Highest, n (%)	73 (39.7)	150 (30.5)

Abbreviations: ENI = Early neurological improvement; IQR = Interquartile range; NIHSS = National Institute of Health Stroke Scale; SBP = systolic blood pressure; SD = Standard deviatio; DBP = diastolic blood pressure; OTT = onset to treatment time; sICH = symptomatic intracerebral hemorrhage; ICH = intracerebral hemorrhage; FBG = fasting blood glucose; TG = triglyceride; TC = total cholesterol; LDL = low-density lipoprotein; TyG = Triglyceride-glucose

Table S9. Association between TyG index and ENI

	Unadjusted		Model 1		Model 2		Model 3		Model 4		Model 5	
	OR (95% CI)	p value	OR (95% CI)	p value	OR (95% CI)	p value	OR (95% CI)	p value	OR (95% CI)	p value	OR (95% CI)	p value
TyG index	0.78 (0.61-0.99)	0.04	0.73 (0.56-0.94)	0.015	0.74 (0.57-0.94)	0.015	0.76 (0.59-0.97)	0.025	0.76 (0.59-0.97)	0.027	0.74 (0.57-0.95)	0.019
**TyG tertiles**		0.079		0.022		0.049		0.065		0.061		0.052
Lowest	Ref		Ref		Ref		Ref		Ref		Ref	
Medium	0.98 (0.64-1.51)		1.00 (0.63-1.58)		0.96 (0.62-1.47)		0.95 (0.62-1.47)		0.97 (0.63-1.50)		0.98 (0.63-1.54)	
Highest	0.66 (0.44-1.00)		0.59 (0.38-0.92)		0.63 (0.41-0.95)		0.64 (0.42-0.97)		0.64 (0.42-0.97)		0.63 (0.41-0.97)	
TyG binary classification												
Lowest to medium	Ref		Ref		Ref		Ref		Ref		Ref	
Highest	0.67 (0.47-0.95)	0.024	0.59 (0.41-0.86)	0.006	0.64 (0.45-0.92)	0.014	0.65 (0.46-0.94)	0.020	0.65 (0.45-0.93)	0.018	0.63 (0.44-0.92)	0.015

Model 1=adjusted for age, sex, initial NIHSS score, Current smoker, atrial fibrillation, SBP, DBP, OTT;

Model 2=adjusted for the minimally sufficient adjustment sets using a directed acyclic graph: age, sex, current smoker, regular alcohol user;

Model 3=adjusted for age, sex, regular alcohol user, previous stroke, coronary artery disease and statin use;

Model 4=adjusted for age, sex, hypertension, atrial fibrillation, coronary artery disease, initial NIHSS score;

Model 5=adjusted for age, sex, initial NIHSS score, current smoker, atrial fibrillation, SBP, DBP, OTT, regular alcohol user, previous stroke, coronary artery disease and statin use;

Abbreviations: TyG = Triglyceride-glucose; ENI = Early neurological improvement; OR = odds ratios; CI = confidence interval; NIHSS = National Institute of Health Stroke Scale.

Table S10. Association between TyG index and ENI by limited to patients who did not receive thrombectomy treatment

	**Unadjusted**		**Model 1**		**Model 2**		**Model 3**		**Model 4**		**Model 5**	
	OR (95% CI)	p value	OR (95% CI)	p value	OR (95% CI)	p value	OR (95% CI)	p value	OR (95% CI)	p value	OR (95% CI)	p value
TyG index	0.81 (0.63-1.05)	0.114	0.75 (0.57-0.99)	0.042	0.78 (0.60-1.01)	0.062	0.79 (0.61-1.02)	0.076	0.79 (0.60-1.03)	0.076	0.78 (0.60-1.03)	0.079
**TyG tertiles**		0.229		0.08		0.184		0.178		0.164		0.203
Lowest	Ref		Ref		Ref		Ref		Ref		Ref	
Medium	0.87 (0.56-1.40)		0.96 (0.59-1.56)		0.88 (0.55-1.39)		0.85 (0.54-1.35)		0.88 (0.56-1.41)		0.92 (0.57-1.49)	
Highest	0.69 (0.44-1.07)		0.62 (0.38-0.99)		0.66 (0.42-1.04)		0.66 (0.42-1.03)		0.65 (0.41-1.03)		0.67 (0.42-1.08)	
**TyG binary classification**												
Lowest to medium	Ref		Ref		Ref		Ref		Ref		Ref	
Highest	0.73 (0.50-1.06)	0.101	0.70 (0.47-1.04)	0.079	0.71 (0.48-1.04)	0.079	0.71 (0.49-1.04)	0.08	0.70 (0.47-1.03)	0.067	0.70 (0.47-1.04)	0.079

Model 1=adjusted for age, sex, initial NIHSS score, Current smoker, atrial fibrillation, SBP, DBP, OTT;

Model 2=adjusted for the minimally sufficient adjustment sets using a directed acyclic graph: age, sex, Current smoker, regular alcohol user;

Model 3=adjusted for age, sex, regular alcohol user, previous stroke, coronary artery disease and statin use;

Model 4=adjusted for age, sex, hypertension, atrial fibrillation, coronary artery disease, initial NIHSS score;

Model 5=adjusted for age, sex, initial NIHSS score, current smoker, atrial fibrillation, SBP, DBP, OTT, regular alcohol user, previous stroke, coronary artery disease and statin use;

Abbreviations: TyG = Triglyceride-glucose; ENI = Early neurological improvement; OR = odds ratios; CI = confidence interval; NIHSS = National Institute of Health Stroke Scal; SBP = systolic blood pressure;,DBP = diastolic blood pressure, OTT = onset to treatment time; ASCO: A (atherosclerosis) - S (small vessel disease) - C (cardioembolic)- O (other causes).

Table S11. Baseline characteristics in patients with and without early sICH

Variable	With sICH (n=28)	Without sICH (n=648)	P Value
**Demographic characteristics**			
Age, y, median (IQR)	73 (68-80)	68 (59-75)	0.003
Sex, n (%)			0.447
Male, n (%)	16 (57.1)	418 (64.2)	
Female, n (%)	12 (42.9)	232 (35.8)	
**Vascular risk factors**			
Current smoker, n (%)	4 (14.3)	194 (29.9)	0.075
Regular alcohol user, n (%)	2 (7.1)	60 (9.3)	0.704
Previous stroke, n (%)	4 (14.3)	98 (15.1)	0.903
Hypertension, n (%)	22 (78.6)	426 (65.7)	0.160
Diabetes, n (%)	12 (42.9)	130 (20.1)	0.004
Dyslipidemia, n (%)	5 (17.9)	202 (31.2)	0.134
Atrial fibrillation, n (%)	20 (71.4)	190 (29.3)	<0.001
Coronary artery disease, n (%)	6 (21.4)	75 (11.6)	0.202
**Medication use history**			
Previous antiplatelet, n (%)	3 (10.7)	72 (11.1)	1.000
Previous anticoagulants, n (%)	1 (3.6)	13 (2.0)	0.450
Previous statin, n (%)	1 (3.6)	49 (7.6)	0.674
Previous antihypertension, n (%)	10 (35.7)	254 (39.2)	0.711
Previous hypoglycemic agents, n (%)	7 (25.0)	74 (11.4)	0.062

**Clinical assessment**			
Initial NIHSS score, median (IQR)	12 (9-17)	6 (3-12)	<0.001
Discharge NIHSS score, median (IQR)	16 (9-36)	2 (1-6)	<0.001
SBP, mmHg, mean±SD	152±30	149±22	0.882
DBP, mmHg, median (IQR)	85 (77-99)	89 (80-98)	0.696
OTT, minute ,median (IQR)	163 (120-200)	180 (120-210)	0.467
Thrombectomy treatment, n (%)	12 (42.9)	81 (12.5)	<0.001
**ASCO Stroke subtype**			<0.001
Atherosclerosis, n (%)	8 (28.6)	242 (37.3)	
Cardioembolic, n (%)	19 (67.9)	192 (29.6)	
Small vessel disease, n (%)	1 (3.6)	69 (10.6)	
Other causes, n (%)	0 (0)	145 (22.4)	
**Laboratory data**			
FBG, mg/dl, median (IQR)	143.3 (11.2-204.2)	101.6 (88.3-122.1)	<0.001
TG, mg/dl, median (IQR)	86.3 (64.6-127.9)	100.1 (70.0-145.3)	0.287
TC, mg/dl, median (IQR)	146.2 (181.0-204.8)	173.2 (143.9-200.5)	0.485
LDL, mg/dl, median (IQR)	121.4 (94.9-142.3)	113.3 (87.0-138.8)	0.222
TyG index, mean±SD	8.87±0.69	8.61±0.70	0.064
**TyG tertiles**			0.030
Lowest, n (%)	9 (32.1)	217 (33.5)	
Medium, n (%)	4 (14.3)	223 (34.4)	
Highest, n (%)	15 (53.6)	208 (32.1)	

Abbreviations: sICH = symptomatic intracerebral hemorrhage; IQR = Interquartile range; NIHSS = National Institute of Health Stroke Scale; SBP = systolic blood pressure; SD = Standard deviatio; DBP = diastolic blood pressure; OTT = onset to treatment time; FBG = fasting blood glucose; TG = triglyceride; TC = total cholesterol; LDL = low-density lipoprotein; TyG = Triglyceride-glucose

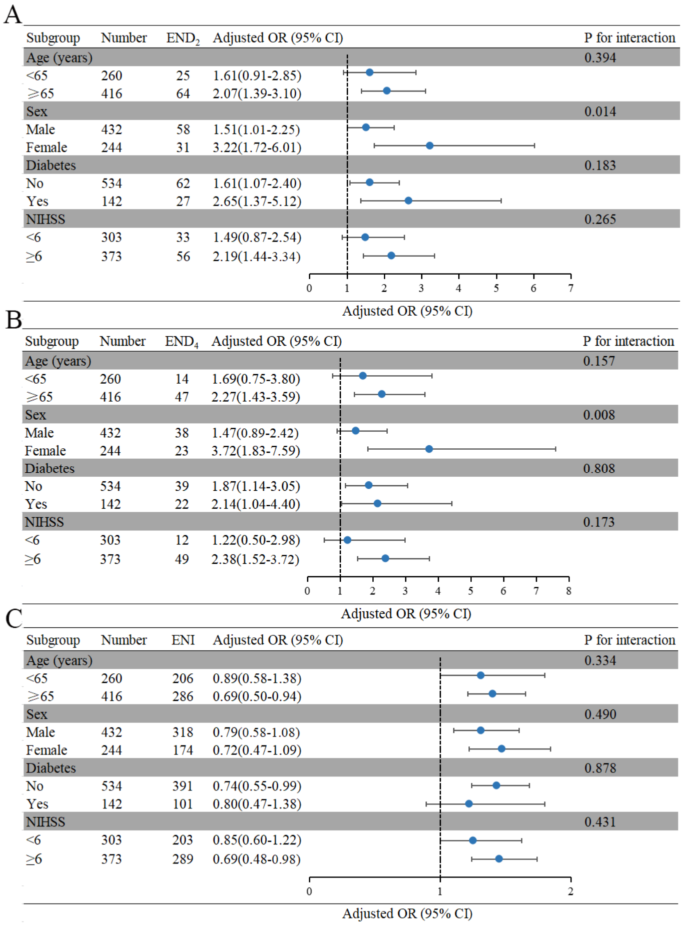

Figure S1. Interactions between the TyG index and END/ENI. Figure legend: A: END2; B: END4; C: ENI;

Adjusted for age, sex, hypertension, atrial fibrillation, coronary artery disease, initial NIHSS score.

Abbreviations: END = Early neurological deterioration; ENI = Early neurological improvement; TyG = Triglyceride-glucose; NIHSS = National Institute of Health Stroke Scale; OR = odds ratios; CI = confidence interval;
